# Extended exergy accounting of agricultural resources in China’s four provinces of mountains and rivers

**DOI:** 10.1038/s41598-025-06828-7

**Published:** 2025-07-01

**Authors:** Hai Qi, Zhiliang Dong, Xinshang You, Shumei Zhou, Yuehong Zhu

**Affiliations:** 1 School of Economics and Management, Hebei University of Science and Technology, Shijiazhuang, 050018 Hebei People’s Republic of China; 2 Hebei University of Water Resources and Electric Engineering, Cangzhou, 061001 Hebei People’s Republic of China; 3 Hebei Key Laboratory of Optoelectronic Information and Geo-detection Technology, Hebei GEO University, Shijiazhuang, 050031 Hebei People’s Republic of China; 4 Hebei University of Science and Technology, Shijiazhuang, 050018 People’s Republic of China

**Keywords:** Resource accounting, Regional agriculture, Extended exergy, Environmental economics, Sustainability

## Abstract

The agricultural sector in China has to balance resource investment, environmental emissions and yields to satisfy the high dietary demands of the population. In this study, extended exergy accounting (EEA) was conducted to analyse the usefulness of resources invested in agriculture and the yields of strategic importance to China in Hebei, Shanxi, Shandong and Henan (four provinces of mountains and rivers) in 2017 from a thermodynamics perspective. The fluxes resources of energy, materials, environmental emissions, labour, capital and yields were measured in joules. The results revealed that the exergy from the natural environment, with an energy exergy value of 9.92 EJ, contributed the most to the agricultural sector in the region. The labour and capital exergy values were much lower than those of the other resources. The non-renewable resource inputs were exceeded by emissions. Animal wastes accounted for 80% of emissions. The resource conversion efficiency in Shandong was 0.46, which was higher than those in the region and China. Shanxi exhibited considerable potential to increase its resource conversion efficiency. EEA was applied in agroecological systems to diagnose the shortage of resource depletion and environmental emissions which could be adopted in other zones or sectors from sustainable development perspective.

**Supplementary Information:**

The online version contains supplementary material available at 10.1038/s41598-025-06828-7.

## Introduction

Agricultural production is highly important for China with large population. Progress has been made in supporting its large population and China encompasses 7% of the total arable land worldwide^1^. The sustainable utilization of resources in agricultural production is from different perspectives, such as energy viewpoint: efficiency^2^, consumption^3,4^, structure^5^; carbon neutrality^6^ and emissions^7^; the production of maize straw^8^ and crop residues^9^; wheat production^10^, and water consumption^11^. In relevant studies, sustainable utilization of resources has been analysed from only one perspective, thus failing to obtain results from an overall perspective. To improve the utilization of resources in agriculture, it is necessary to account the usefulness of different resources in overall perspective from thermodynamics view.

Exergy characterizes the largest amount of work that can be extracted from one system or process^12^. Exergy analysis results can exhibit the potential usefulness of exergy in solving environmental problems and moving to sustainable development^13^. Compared with energy analysis, or resource accounting by currency or mass, exergy represents both the quality and quantity of resources^14^. Since exergy unifies different resources into joules, exergy accounting can facilitate ecological diagnoses and provide the determination of the development potential from sustainability^12^. A higher exergy value indicates greater potential to influence the environment^15^.

Exergy analysis has been applied in different sectors and countries to increase the efficiency of resource use. It has been applied in the agricultural sectors of China^16–18^, Iran^19^ and Belgium^20^. Moreover, exergy analysis has been applied in the transportation systems in China^21–24^, Turkey^25–27^ and Greece^28^, as well as in other sectors, such as the industrial sectors in Denmark^29^ and Turkey^30^, rose planting^31^, smelting and pressing of metals industry in China^32^ and the energy sector in Mexico^33^. The concept of the exergy flux was subsequently elevated from the sector scale to the country or regional scale: countries in Asia (India^34^, China^35,36^ and Singapore^37^); Europe (Spain^38^, Greece^39^ and the UK^40^); the United States^41^. On the basis of these studies, the exergy flux situation was compared among countries^42–44^. These studies can be grouped into three types. First, only energy resources were analysed in Saudi Arabia^45^; Second, energy and material consumption resources were analysed: in Norway^46^, China^47^ and the whole world^48^; Third, remediation costs, capital and labour were added to exergy accounting in Italy^49^, Turkey^50^ and China^51,52^. Exergy analysis shows the advantages of accounting for resource conversion, thereby revealing improvements in sustainable development levels and providing suggestions^53^. Exergy is measurement of ecological complexity regarding how far the observed ecosystem from reference environment^13^. It is employed to reflect the ecosystem resource availability and environmental impacts^54^. Extended exergy accounting (EEA) has expanded the study scope of exergy analysis, including energy, materials, labour, capital and environmental costs, making it more meaningful and simpler to evaluate resource utilization and production (expressed in joules)^55^. Moreover, the approach crosses the social, economic and natural environments, reflecting the “ecological cost” of resources in one system. EEA connects thermodynamics, ecological and economic costs in the estimation of the depletion of different resources from a sustainable development perspective^56^.

Facing a large population, a shortage of arable land and an upgrading food structure, China has to increase its agricultural production efficiency. Hebei, Shanxi, Shandong and Henan Provinces (four provinces of mountains and rivers, FPMR) are located in northern China, the lower reaches of the Yellow River and exhibit continental monsoon climate. These provinces are close to Beijing and Tianjin, which are among the most developed regions in China. In 2017, the FPMR accounted for 22.15% of the total population, yielding 24.85% of the total grains and 31.11% of the total vegetables in China. The population density was 2.18 times greater than that of China as a whole. Agriculture is critical for the region as it encompasses corn, wheat and vegetable production areas. Chinese agriculture was performed in exergy analysis^18,57,58^, without GHG emissions accounted for, and the sector transitioned from relying on renewable resources to non-renewable resources^17^. The extended exergy accounting approach was subsequently applied to agricultural production in Hebei Province^59^. However, previous studies lacked comprehensive description of regions larger than province scale, such as the FPMR, which is close to Beijing and Tianjin. Furthermore, it is worth analysing the agricultural features of several provinces from an exergy perspective and comparing them with those of China for sustainable development. Therefore, sustainable development of agriculture in areas should still be explored comprehensively.

**Fig. 1:**
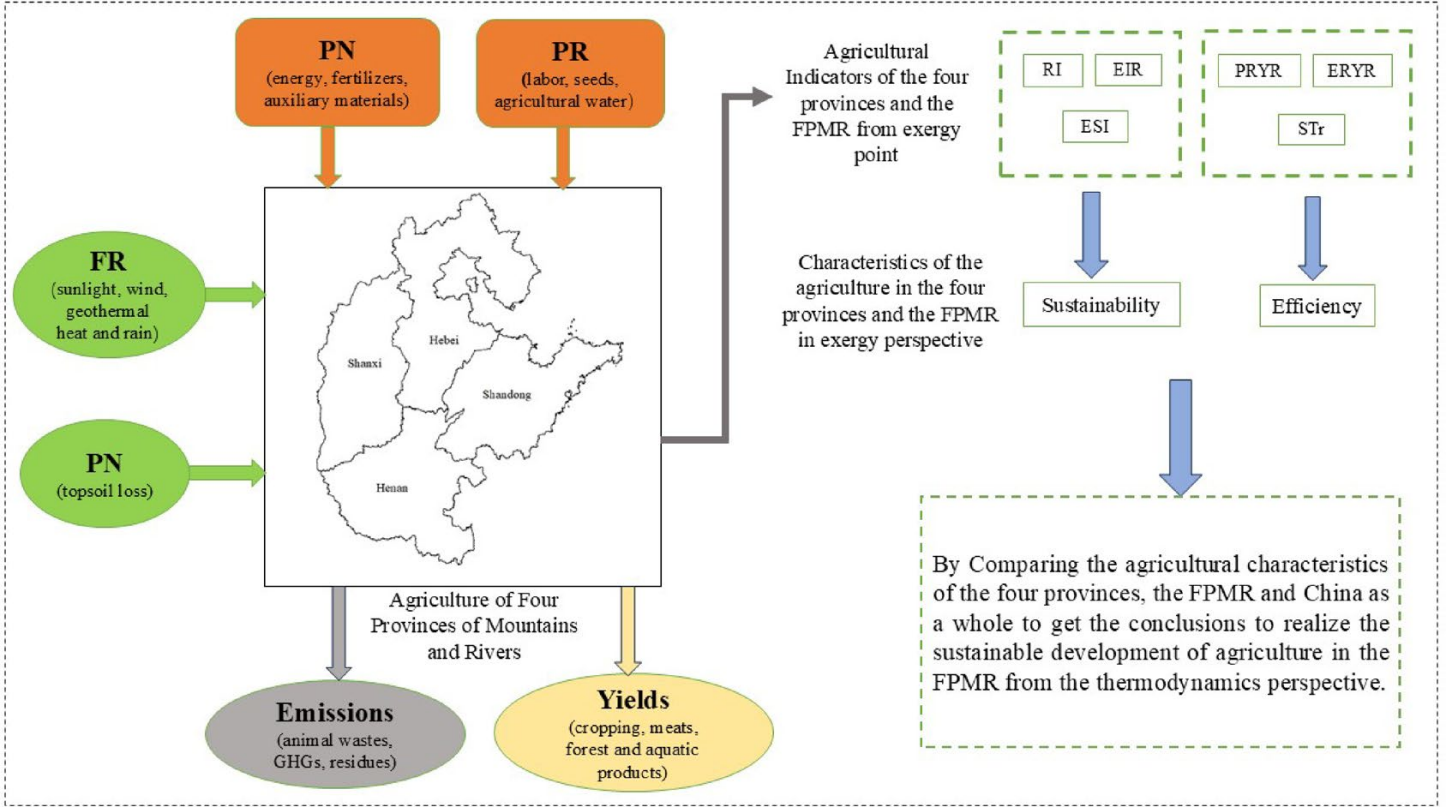
Roadmap.

To fill this gap, the EEA method was employed to evaluate the resource utilization status in the FPMR in 2017 from a thermodynamic perspective. The inputs of energy, materials, labour, capital, yields, emissions and evaluation indices for the exergy flux in the agricultural sector were analysed, as shown in Fig. 1. This approach could be used to optimize resource use^60^ for sustainable development of agriculture in other zones or industries.

## Methods

The exergy resources in agricultural system could be classified into four categories^17,18^: free renewable resources (FR) (sunlight, wind, rain and geothermal heat); purchased renewable resources (PR) (agricultural water (A_water_), labour and seeds); free non-renewable resources (FN) reflecting topsoil loss and erosion; and purchased non-renewable resource (PN) (coal, diesel, gasoline, natural gas, electricity, agricultural machinery, plastic mulch, pesticides and chemical fertilizers). The yield (Y) includes the production of cropping, stockbreeding, forestry, fishery and straws. The environmental remediation cost (E_R_) refers to residues of chemical fertilizer, pesticides, plastic mulch and animal waste^18^. The exergy of greenhouse gases (GHG) has been estimated in different sectors in China^14^, and these emissions were incorporated in the environmental remediation cost.

E_E,_ E_M_, E_K_, E_L_ and E_R_ are the exergy equivalents of energy, material, capital flows, human labour and remediation cost expressed in joules^13,22,56^, respectively. The parameter evaluations are listed in Table 1. f is the ratio of HDI to HDI_0_, where the HDI (human development index) represents the standard of life in different regions or countries published by the United Nations; e_surv_ is10^7^ J, which represents the necessary exergy consumption of one person in one day; N_h_ indicates the Chinese population in 2017; and deposits constitute a large part of M2 in China, which is different from the western financial system. W, s and N_w_ are the average workload (2000 h), average wage and number of workers, respectively.

**Table 1:** The parameters evaluation.

Parameter	Unit	Value (for China, 2017)
f	–	0.75/0.055
e_surv_	J/(personday)	10^7^ J/(person × day)
N_h_	Population	1.4 × 10^9^
M2	RMB/year	GDP (8.2 × 10^13^ RMB)
s	RMB/year	7.4 × 10^4^
N_w_	Population	7.9 × 10^8^
W	Workhours/(person × year)	2000 h/(person × year) = 8 h/day × 250 day/year

E_L_ and E_K_ are expressed as follow:

1 $${\text{E}}_{{\text{L}}} = {\text{ f}} \times {\text{e}}_{{{\text{surv}}}} \times {\text{N}}_{{\text{h}}}$$

2 $${\text{E}}_{{\text{K}}} = \frac{{{\text{M}}2}}{{{\text{s}} \times {\text{N}}_{{\text{w}}} \times {\text{W}}}} \times {\text{E}}_{{\text{L}}}$$

The exergy equivalents of capital and labour were determined for the four provinces, with data extracted from the China Labour Statistical Yearbook^61^. First, we calculated the E_L_ value of Chinese society. Next we obtained the E_L_ value in the agricultural sector of the four provinces based on the number of workers and their wages^56^. Finally, we computed the E_K_ value of agriculture in the region. Following previous studies^13,56^, GDP was employed as an indicator to represent monetary circulation over 1 year. The detailed calculations of E_L,_ E_K_ and E_R_ are provided in the Supplementary Information.

Exergy provides a unified metric for resource depletion in the production process; therefore, it is easier to compare and calculate the input–output structure^18^. Renewability index (RI) is the total renewable resources divided by the total non-renewable resources, with a lower value implying greater resource stress to the ecological environment. Purchased resource yield ratio (PRYR) is the ratio of the yield (Y) to the purchased resources, with a higher value representing higher payback per unit input. Economic investment ratio (EIR) is the purchased input divided by the free natural resource inputs, with a smaller value suggesting a lower stress of economic investment. Environmental resource yield ratio (ERYR) is calculated as the yield divided by the sum of free environmental resource investment and the environmental remediation cost, indicating the influence of free resources and the environmental cost in the production system. A larger value indicates that fewer environmental resources are required to obtain one unit of yield. Environmental stress index (ESI) represents the stress of agricultural activities on the environment and is measured by the ratio of free resources, agricultural water and the environmental remediation cost to the purchased input, excluding agricultural water. System transformity (STr) reflects the efficiency of agricultural production and is measured as the yield divided by all inputs, including free resources, purchased resources and environmental remediation costs.

3 $${\text{Renewability index }}\left( {{\text{RI}}} \right) = ({\text{FR}} + {\text{PR}}){/}({\text{FN}} + {\text{PN}})$$

4 $${\text{Purchased resource yield ratio }}\left( {{\text{PRYR}}} \right) = {\text{Y/}}({\text{PR}} + {\text{PN}})$$

5 $${\text{Economic investment ratio }}\left( {{\text{EIR}}} \right) = ({\text{PR}} + {\text{PN}}){/}({\text{FR}} + {\text{FN}})$$

6 $${\text{Environmental resource yield ratio }}\left( {{\text{ERYR}}} \right) = {\text{Y/}}({\text{E}}_{{\text{R}}} + {\text{FR}} + {\text{FN}})$$

7 $${\text{Environmental stress index }}\left( {{\text{ESI}}} \right) = ({\text{FR}} + {\text{FN}} + {\text{A}}_{{{\text{water}}}} + {\text{E}}_{{\text{R}}} ){/}({\text{PR}} + {\text{PN}} - {\text{A}}_{{{\text{water}}}} )$$

8 $${\text{System transformity }}\left( {{\text{STr}}} \right) = {\text{Y/}}({\text{FR}} + {\text{FN}} + {\text{PR}} + {\text{PN}} + {\text{E}}_{{\text{R}}} )$$

The exergy coefficients of different resources have been estimated in previous studies^14,52^, as listed in Table Supplementary. The yields data were extracted from the China Statistical Yearbook^62^, China Agriculture Yearbook^63^ and China Forestry Yearbook^64^; labour and capital data were from China Labour Statistical Yearbook^61^; energy consumption data came from China Energy Statistical Yearbook^65^.

## Results

### Investment

**Fig. 2:**
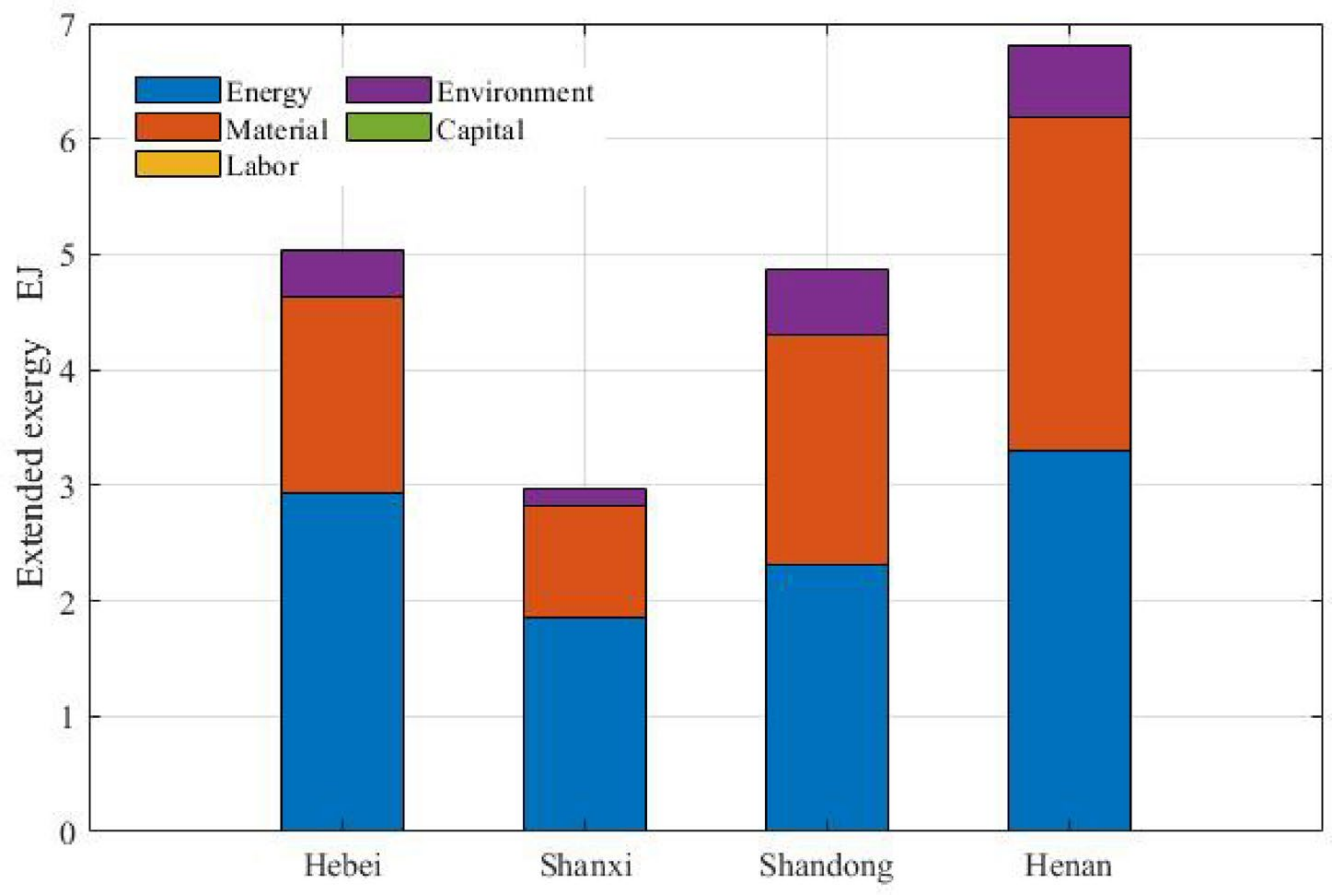
Extended exergy structure.

Figure 2 shows the extended exergy components in the region, with Henan ranking first and Shanxi ranking last. The energy exergy value in Henan was 3.30 EJ greater than the extended exergy investment in Shanxi (2.95 EJ). Energy exergy was the largest contributor and material exergy was the second largest contributor. The proportions of environmental remediation, labour and capital in the exergetic values were much lower than those of energy and materials, with capital as the least important component in each province. The extended exergy inputs in Henan accounted for 34.63% of those in the area, with the exergy equivalents of energy, material and environmental remediation accounting for the greatest shares within the region. The shares of extended exergy from Hebei, Shandong and Shanxi were 25.57%, 24.76% and 15.04%, respectively. The exergy equivalent of labour and capital in Shandong were the highest. Located in northern China, Hebei and Shanxi exhibit greater proportions of forest areas (26.91% and 27.44%, respectively), preventing the occurrence of sand and dust storms. Planting trees is a difficult task with low short-term economic interest. Hebei and Shandong accounted for 25.41% and 22.34% of the FR. The exergy of topsoil loss and erosion (FN) in the region corresponded to 0.70% of the FR and 9.11% of the non-renewable investments, according to previous study^18^.

The purchased non-renewable resources (PN) involving energy, fertilizers and auxiliary materials (pesticides, plastic mulch and agricultural machinery) are essential in modern agriculture. The PN in Shandong accounted for 30.05% of the total energy, and Henan accounted for 38.01% of the total auxiliary materials and 43.80% of the total fertilizer. The proportion of energy accounted for the highest percentage in Hebei (51.78%), Shanxi (64.67%), Shandong (42.19%) and auxiliary materials ranked second. In contrast, auxiliary materials accounted for 37.32% and energy accounted for 33.53% of the PN in Henan. Fertilizer accounted for the smallest proportion in the four provinces.

The energy values in Shandong and Shanxi were 148.38 and 75.63 PJ, respectively. In Shandong, diesel and gasoline accounted for 62.52% and 1.79% of energy inputs, with electricity accounting for 26.70% and coal accounting for 8.99%. Similar situation was observed in Hebei and Henan. Shanxi consumed 38.48 PJ of coal, accounting for 50.88% of the energy exergy; the consumption of oil accounted for 29.53%, and electricity attained the lowest percentage. Hebei consumed 52.80% of the total amount of gasoline and 50.82% of the total amount of natural gas, Shanxi consumed 46.78% of the total amount of coal, and Shandong consumed 43.19% of the total amount of diesel and 31.68% of the total electricity. Mechanical equipment dominated the auxiliary materials, accounting for 83% in the four provinces on average. Pesticides and plastic mulch ranked the second and third, accounting for 11.30% and 5.70%, respectively, on average. Nitrogen accounted for the largest part of fertilizer use in Hebei (61.04%), Shandong (47.22%) and Henan (47.35%), with compound fertilizer accounting for the second largest part, followed by phosphate and potash. In Shanxi, the most common fertilizer was compound fertilizer (49.07%), followed by nitrogen (39.40%), phosphate and potash. Henan consumed the majority of the four types of fertilizers. The inputs of fertilizers and mechanical equipment per unit of arable land in the FPMR were greater than those in China. Agricultural water occupied the greatest part of the PR being the largest water expenditure in the region, Hebei (69.44%), Shanxi (60.75%), Shandong (63.96%), and Henan (52.52%), while the percentage in China as a whole was 62.32%.

Human labour comprises of cropping, stockbreeding, forest, fishery and services. In Hebei, the labour exergy value was 4.29 PJ, and cropping accounted for the largest proportion (32.52%), followed by services (31.49%), forestry (25.59%), stockbreeding (9.69%) and fisheries (0.71%). The labour in services accounted for the highest proportion in others. The smallest contributor to labour was fisheries in Hebei, Shanxi and Henan, whereas stockbreeding was the smallest contributor in Shandong. Hebei, Shanxi, and Henan contributed the most to cropping (36.67%), forestry (38.32%), and stockbreeding (46.33%) respectively. Shandong accounted for the highest percentage of fisheries (69.43%) and services (37.25%). The share of labour in the region was smaller than that in China in terms of cropping, whereas the shares of the other four items were greater than those in China. In particular, the share of services was 1.67 times greater than that in China. Machines could compensate for the work performed by female labour. Females accounted for 33.91% of the labour force in Hebei, being greater than that in the other provinces and the FPMR (31.56%), less than that in China (35.09%). Shandong attained the lowest female labour ratio. The exergetic value of capital was much lower than that of labour. The exergy equivalents of labour and capital were much lower than those of the other components, indicating that labour and capital were not the key factors in modern agriculture. The purchased non-renewable resource inputs were more important than the labour and capital inputs from an exergy perspective.

### Emissions

**Fig. 3:**
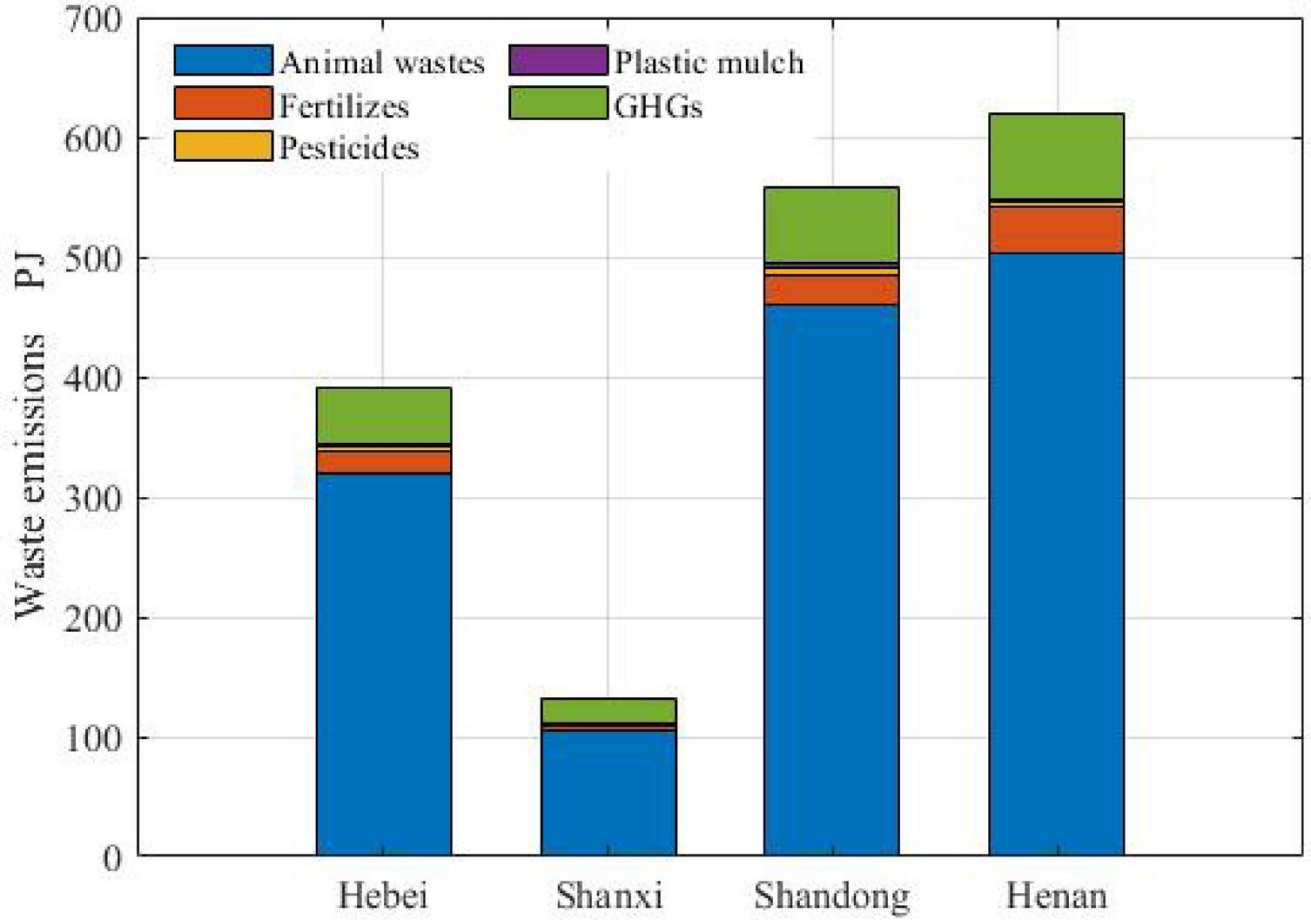
Emissions.

Animal waste constituted 80% of the total emissions, followed by GHG emissions, accounting for approximately 11% of the total emissions, as shown in Fig. 3. The residues of fertilizers, pesticides and plastic mulch accounted for the remaining part. Henan accounted for the largest proportion of emissions (36.45%), 36.28% of animal waste, 35.14% of GHG emissions and 43.80% of fertilizer residues. The highest percentages of pesticide residues (38.25%) and residues from plastic mulch (46.12%) were observed in Shandong. In Hebei and Shanxi, waste from large animals (horses, donkeys, cattle and mules) constituted the largest proportion (36.49% and 33.67%, respectively), followed by that from hogs, poultry and sheep. In Shandong and Henan, the percentages of animal waste from hogs were 36.56% and 48.31%, respectively, followed by those from large animals, poultry and sheep. Waste from hogs accounted for the largest proportion of animal waste.

The exergy of GHG emissions (CO_2_, N_2_O and CH_4_) in agricultural activities was estimated based on previous studies^14,18^. Methane emissions come from grain production, livestock and fossil energy use; carbon dioxide emissions are produced from fossil energy use; and nitrous oxide emissions are derived from the application of nitrogen fertilizer, fossil energy, and livestock. Methane emissions contributed the most to the total GHG emissions, followed by carbon dioxide and nitrous oxide emissions. Grain production contributed the most to methane emissions (65.39%), followed by livestock (33.90%) and fossil energy use (0.71%). Compared with China, the proportions were 53.66%, 45.27% and 1.07%, indicating that the level of grain production in the region was greater than the average level of China.

Henan accounted for the greatest percentages of GHG (35.14%), methane (35.72%) and nitrous oxide emissions (39.36%), and Shandong owned the greatest percentage of carbon dioxide emissions (29.56%). Among the four GHG sources, grain production accounted for 61.61% of the total GHG emissions, followed by livestock (32.00%), energy use (6.31%) and nitrogen fertilizer (0.08%). The corresponding average percentages in China were 50.99%, 43.07%, 5.87% and 0.07%, respectively, indicating that the proportion of livestock in the region was smaller than that in China. Shandong accounted for the majority of GHG emissions from energy (28.35%) and livestock (31.99%), and Henan accounted for the majority of the GHG emissions from nitrogen fertilizer (41.69%) and grain (38.19%). Although the levels of residues of fertilizers, pesticides and plastic mulch were lower than those of the other factors, excessive application can lead to soil compaction, a decline in arable land productivity and other problems.

### Yields

**Fig. 4:**
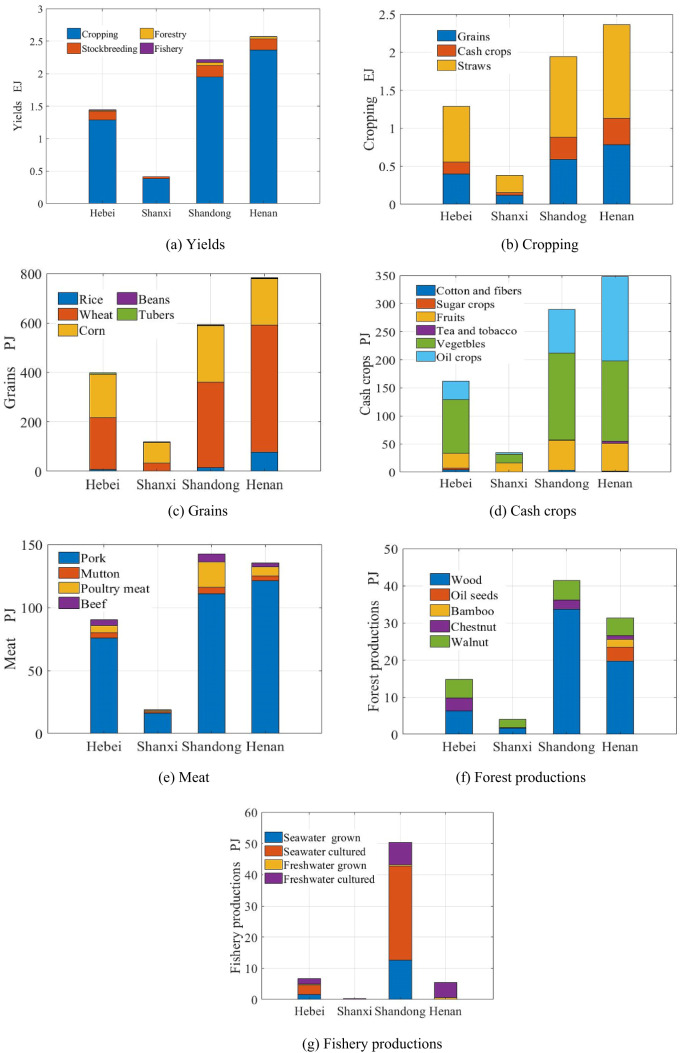
Yields of agriculture.

Henan exhibited the highest percentage of crops (39.53%) and yields (38.69%), while Shandong exhibited the highest percentages of stockbreeding (35.29%), forestry (45.24%) and fishery (80.05%), with the second highest yield (33.37%), as shown in Fig. 4a. Cropping dominated the yields, followed by stockbreeding, forestry and fishery, except Shandong owning lots of fishery yields.

Figure 4b shows that the cropping system contained three parts: grains, cash crops and straw. In terms of inevitable production, straw accounted for more than half of the yield (54.36%), followed by grains (31.67%) and cash crops (13.97%). Henan led in terms of grains (41.36%), cash crops (41.69%) and straw (37.91%) in the region. Grains included rice, wheat, corn, soybean and tubers as shown in Fig. 4c. Located in water shortage area, wheat and corn dominated grain production (93.88%). Unlike in the other three provinces, the exergy of corn was greater than that of wheat in Shanxi. In China, rice constituted the largest proportion of grains (44.10%), followed by corn (29.24%) and wheat (24.51%). Henan accounted for several of the highest shares: rice (77.47%), wheat (46.68%), soybeans (39.20%) and grains (41.36%); Shandong and Hebei accounted for the largest shares of corn (33.93%) and tubers (38.25%). Cash crops were divided into six subgroups; vegetables, oil crops, cotton and fibres, tea and tobacco, fruits and sugar crops, as displayed in Fig. 4d. Located close to Beijing and Tianjin, the FPMR has aimed to produce vegetables and fruits to supply these cities, with 66.29% of the total cash crops, 30.86% of the total vegetables and fruits in China. In particular, the cash crop ratios of vegetables from Hebei and Shandong were 59.36% and 53.26%, respectively. The exergy of oil crops was lower than that of vegetables and higher than that of fruits in the region, with the proportion of oil crops reaching 31.68% of the cash crops. Peanut accounted for 88.12%, although the oil crops included rape seeds, sunflower seeds, sesame and flaxseed. Cotton and fibre, tea and tobacco and sugar crops constituted small fractions. The crop/straw ratios for cotton, sesame, soybean, rapeseed, maize, wheat, rice, peanut, tubers, sugarcane, vegetables and beet were estimated at 9.2, 2.2, 1.6, 1.5, 1.2, 1.1, 0.9, 0.8, 0.5, 0.24, 0.1 and 0.08, respectively^18^. The percentage of straw from wheat and maize was 80.34% in the region. Notably, the proportion of wheat and maize in the area was much greater than that in China (52.42%).

Stockbreeding production comprises meat, milk, egg, wool and cashmere, honey and silkworm cocoons. Meat (pork, poultry, mutton and beef), milk and eggs accounted for more than 99% of total stockbreeding production, and meat accounted for 75.24% of the livestock production. Pork constituted the largest part of the meat production, followed by poultry meat, except in Shanxi where pork was followed by mutton, poultry and beef, as shown in Fig. 4e. Shandong accounted for the largest shares of meat (36.82%), mutton (35.73%), poultry (58.80%) and beef (44.03%), while Henan and Hebei exhibited the highest shares of pork (38.25%), milk and eggs (33.72%). Forest products included wood, chestnut, walnut, oil seeds and bamboo as revealed in Fig. 4f. Wood accounted for the largest share (67.08%), followed by walnut and chestnut, except in Shanxi, where walnut was accounted for the largest part, followed by wood and chestnut. In contrast with the other provinces, only Henan produced oil seeds and bamboo. Even so, Shandong accounted for the greatest share of forestry yields (45.24%), wood (54.90%) and walnut (30.30%), while Hebei accounted for the greatest share of chestnut (49.21%). In China the largest contributor was bamboo (82.37%). Fishery production included seawater grown, seawater cultured, fresh water grown and freshwater cultured as shown in Fig. 4g. The cultured aquatic product was the dominant share of the fishery. The fishery yields from seawater were much greater than those from freshwater. Shellfish and fish accounted for 44.12% and 41.12% of the total yield. Shandong monopolized the aquatic production (80.05%). Henan attained the highest percentage of freshwater grown (45.12%).

**Fig. 5:**
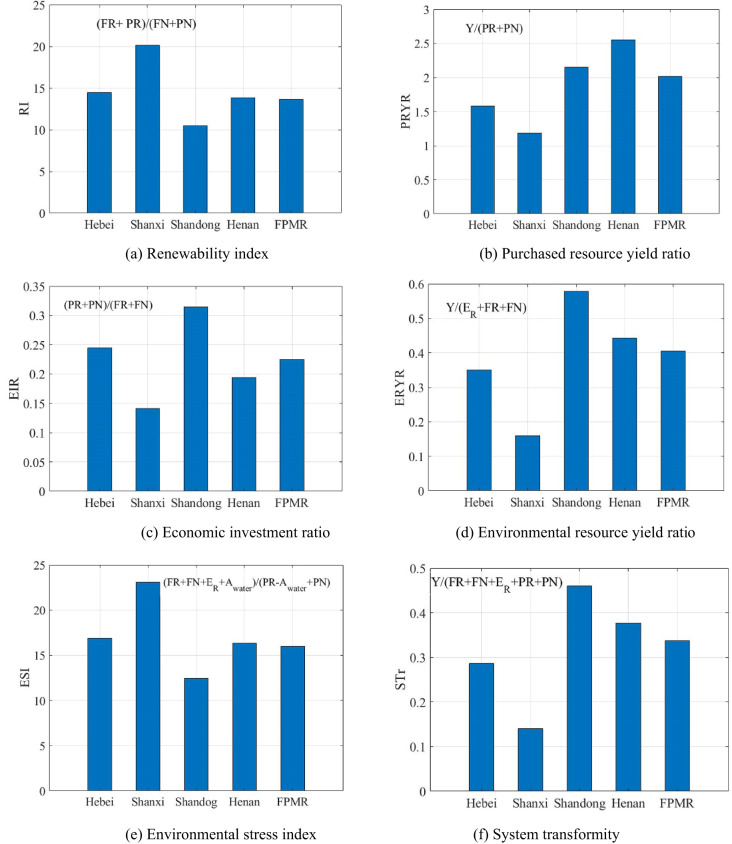
Exergy-based indicators.

The value of the renewability index (RI) in Shanxi was 20.11, more than 47.33% of the regional level as shown in Fig. 5a. It revealed that fewer non-renewable resources with the same amount of renewable resources were invested in Shanxi, indicating that agricultural activity was more sustainable in Shanxi. The PRYR value in Henan reached 2.55, more than 26.73% that of the FPMR, as shown in Fig. 5b. The highest value of the EIR was 0.31 in Shandong, more than 39.83% of that in the region, as shown in Fig. 5c. These findings indicated that the amount of purchased resources was much smaller than the amount of environmental investment. Although the exergetic values of yields, emissions, FR and FN in Shandong were lower than those in Henan, Shandong attained the highest ERYR value, 3.63 times that in Shanxi, as shown in Fig. 5d. To obtain one unit yield, Shanxi depleted the greatest amount of environmental resources. The ESI value in Shanxi was 1.85 times that of Shandong, implying that Shanxi had the least stress as shown in Fig. 5e. Compared with Henan, only the PR value in Shandong was more than 55.12 PJ in terms of FR, FN, PR, PN, emissions and yields. The STr in Shandong was 0.46, greater than 34.85% of that in the region as shown in Fig. 5f.

**Fig. 6:**
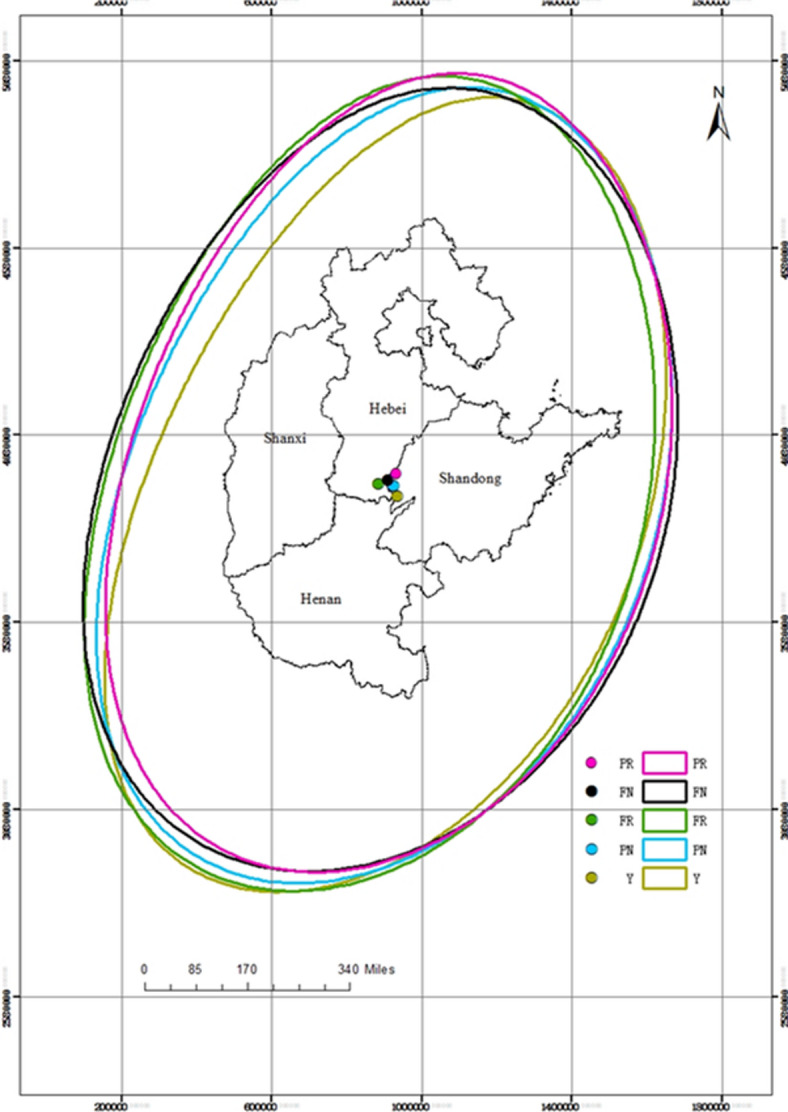
The SDEs of resources.

The standard deviational ellipse was employed to visualize the distribution of the elements, as shown in Fig. 6. It reflects the dispersion status of the elements in spatial patterns^66,67^. The ellipses of FR, FN, PR, PN and yields were distributed in the northeast–southwest direction. The centre of the FR was located nearly in the centre of the region. The yields in Henan and Shandong were much greater than those in Shanxi, leading the centre of yields to move along the southeast direction obviously. The ratio of the long-axis to the short-axis was 1.61, indicating that the dispersion of yields was greater than that of the other components, as revealed in Table 2.

**Table 2:** The parameters of the generated SDEs in the FPMR.

	Longitude	Latitude	Short-axis	Long-axis	Rotation	Long-axis/Short-axis
Yield	115.59	36.17	6.74	10.86	43.69	1.61
PN	115.51	36.41	7.32	10.66	43.97	1.46
FR	115.05	36.50	7.51	10.66	40.35	1.42
FN	115.34	36.57	7.73	10.46	47.11	1.35
PR	115.63	36.71	7.50	10.46	41.61	1.39

To produce 100 units of yield, different investments were needed, as shown in Table 3. Free renewable resources were the greatest contributor to agricultural production. The emission exergy value was greater than the non-renewable resource exergy inputs except in Shanxi. The conversion efficiency in Shanxi ranked at the bottom among its regional counterparts, approximating the overall level observed across China. Agricultural production did not rely on much labour in the region. Especially labour of cropping and livestock in Shandong being only 0.38 PJ and 0.09 PJ yielded 1.9 EJ and 181.2 PJ, respectively, its efficiency much greater than that in the region and China. Being the shortcoming of the region, forest production in Henan was 31.31 PJ with 0.65 PJ of labour, the ratio of which is almost equal to that in China. The input–output efficiencies of cropping and livestock in Shandong were 159.27 and 38.06 Kt/person, respectively, according to mass accounting, much greater than those in the region and China. In this way, the cropping efficiency in Hebei was lower than that in Shanxi, on the contrary of the result from the EEA.

**Table 3:** Resource investment to obtain 100 units yields.

Resource	Hebei	Shanxi	Shandong	Henan	Region	China
FR	256.76	591.85	146.80	200.36	219.23	597.42
FN	1.89	4.11	1.05	1.35	1.54	3.78
PR	44.26	56.16	30.64	24.29	32.73	61.64
PN	18.93	28.11	15.86	14.89	16.92	13.25
Labor	0.30	1.09	0.21	0.15	0.26	1.57
E_R_	27.09	31.60	25.13	24.09	25.56	26.85
Capital	0.00020	0.00075	0.00014	0.00011	0.00018	0.00108

## Discussion

Since the agricultural production area in Henan was much greater than that in Shanxi, Henan absorbed the most exergy whereas Shanxi absorbed the least exergy. Photosynthetic exergy accounted for the highest percentage of FR, followed by rain, wind and geothermal heat. Due to the greater precipitation in Henan, the exergy of rain reached 2.11 EJ, accounting for 41.04% of the FR, which was greater than that in the other provinces in terms of proportion and amount. Land absorbs more photosynthetic exergy than water does, and the land area is much larger than the water area in the region. Grain production was encouraged by the Chinese government with favourable condition to ensure the national food security. The agricultural area in Henan is greater than that in the other regions, and the value of photosynthetic exergy (2.35EJ) is almost equal to the FR in Shanxi (2.46EJ). Except for Shanxi, the cropping area accounted for the largest part of agricultural land. Pastures accounted for 38.91% of agricultural land, with the cropping area (30.58%) being the second largest area in Shanxi. With fewer plains, less rainfall and more mountainous areas, Shanxi attained the lowest FR, accounting for 16.89% of the regional value.

Extended exergy accounting revealed the exergy flux situation in the agricultural sector of the four provinces adjacent to Beijing and Tianjin. Since the yields from cropping dwarfed the other three subindustries, arable land was the key resource for agricultural activities. Water is a vital element in agricultural activities^68^. The per capita water resources in the region reached 14.9 GJ/person, much lower than that in China (103.45 GJ/person). In the area, the percentages of wheat, maize, rice, soybean and tubers were 58.26%, 35.68%, 5.23%, 0.28% and 0.61%, respectively, whereas the percentages were 24.51%, 29.24%, 44.10%, 0.94% and 1.21%, in China. Owing to water shortage, aquatic products from seawater was 3.08 times of that from freshwater. The per capita water resources were 9.20 GJ/person in Hebei, accounting for 52.29% of that in Shanxi. Hebei contains no large river in its territory. To satisfy the water demand, overexploitation of groundwater has become necessary, leading to the occurrence of groundwater funnels. To save water resources, irrigation via dripping and pipelines were applied during cropping^69^. Given the institutional advantages of China, water resources can be allocated from an overall rather than from a partial perspective.

It is difficult to reduce emissions in the agricultural production process. The exergy value of emissions was greater than that of non-renewable resources. Emissions from fossil energy and chemical product consumption were much lower than those from animal waste, which could be converted to organic fertilizer by fermentation. Thus, the capacity of arable land increased and animal waste was managed properly. Agriculture generates large amounts of GHG emissions in China^70^, so does the region.

Dietary requirements, especially animal-based food consumption, affect food production. An obvious per capita dietary difference existed between urban and rural households in the four provinces and China in 2017, as detailed in Table 4. From per capita perspective, urban residents consumed less grain, more animal-based foods and vegetables than did rural households, excluding Henan. In terms of grain consumption, Shanxi exhibited the largest difference between rural and urban areas, reaching 45.1 kg, almost equal to the level across China. The greatest differences in meats, eggs, milk and vegetables were 9.8 kg, 4.0 kg, 16.9 kg and 27.2 kg, in Henan, and the differences were greater than those in China. Shandong exhibited the largest difference in aquatic products, reaching 9.3 kg, greater than the level in China. The urban population accounted for 55.69% of the total population in the region. Therefore, the potential demand for animal-based foods is high. A growing requirement for meat, egg and milk requires greater resource investment and results in higher emissions^71^. Facing problems in terms of the supply and demand for animal-based foods, these four provinces could increase the resources within and outside of the region, for example, by introducing different meats or forages from other areas of China or globally.

**Table 4:** Per capita consumption of foods in the FPMR and China in urban and rural.

	Hebei		Shanxi		Shandong		Henan		Region		China	
	Urban	Rural	Urban	Rural	Urban	Rural	Urban	Rural	Urban	Rural	Urban	Rural
Grain	114.8	134.3	114.3	159.4	111.2	136.6	137.2	136.5	119.8	138.6	109.7	154.6
Meats	28.2	19.1	19.7	12.3	31.0	21.6	26.2	16.4	27.6	18.1	38.9	31.5
Eggs	15.1	13.0	11.5	10.2	17.9	16.3	16.5	12.5	16.0	13.4	10.9	8.9
Milk	20.8	8.3	19.9	9.6	21.9	11.7	23.2	6.3	21.7	8.7	16.5	6.9
Aquatic products	7.9	3.7	3.4	1.2	15.8	6.5	5.2	2.6	9.4	3.8	14.8	7.4
Vegetables	100.4	75.1	88.7	64.9	102.8	82.7	99.3	72.1	99.5	75.1	106.7	90.2

Crop residues and animal wastes can be used to produce more carbon dioxide through fermentation in greenhouses. More carbon dioxide might suffocate certain pests without pesticides and promote the photosynthetic effect on crops. Crop residues might also be utilized as raw materials for fertilizer, forage or other industries^6^. Shandong might strive to develop fisheries along its long coastline, whereas Shanxi might attempt to expand its forestry since it is surrounded by the Taihang and Lvliang Mountains. Agricultural indicators are listed in Table 5 containing the four provinces, the FPMR and China in 2017. Only the ESI and RI values in China were greater than those in the region, whereas the other indices exhibited the opposite. Over the past decade, E_L_ in China increased while it decreased in agriculture and the same situation happened in the region. Although the labour declined, yields increased in China and the FPMR, owning to the continuous non-renewable resource inputs. The agricultural production efficiency in China was lower than that in the area and efficiency in Shanxi was almost equal to that in China, meaning the potential of Shanxi was huge.

**Table 5:** The indices of the FPMR and China.

Index	Hebei	Shanxi	Shandong	Henan	FPMR	China
RI	14.46	20.11	10.49	13.83	13.65	38.71
PRYR	1.58	1.19	2.15	2.55	2.01	1.34
EIR	0.24	0.14	0.31	0.19	0.22	0.12
ERYR	0.35	0.16	0.58	0.44	0.41	0.16
ESI	16.91	23.06	12.47	16.32	15.98	45.69
STr	0.29	0.14	0.46	0.38	0.34	0.14

Since EEA theory measure the degradation of resources in conversion process based on thermodynamic laws, other features of resources are neglected. EEA quantifies the exergy value of labour by the interchange of labour and commodities^51^, unlike other studies that aim to measure labour via monetary methods. In reality, capital contains not only wages but also credit and monetary overspending, which might cause uncertainty in capital flows. The theoretical foundation of exergy analysis for resources is the thermodynamics law, whereas labour and capital represented by exergy do not follow the thermodynamic rules strictly, being a “parallel” cost process with the supply chain of economics^72^. The emissions accounting fails to reflect the poisonousness of the release for the long-term time or space precisely. It lacks the ability to show environmental influences comprehensively^73^. Quantifying the efficiency of labour reasonably may be a challenge of EEA in the future^74^.

It is difficult to increase agricultural yields only by expanding investments, therefore, a reasonable alternative is to increase efficiency of inputs^75,76^. In this study, EEA and indices were applied to estimate the resource utilization in the agricultural sector within the area from a thermodynamic perspective. EEA unifies a variety of resources from a sustainable development perspective, which can be applied to increase the sustainable development of in other areas or sectors.

## Conclusions

With its large population, China has to ensure national food security and sustainable agricultural development, and the FPMR faces similar challenges. From a thermodynamic perspective, cropping production accounts for the largest proportion of yield in the region. These four provinces could focus on different aspects in future agricultural production. Hebei could develop water-efficient cropping continuously while prioritizing supply to Beijing and Tianjin. With inferior agricultural conditions compared to the other three provinces and production level close to the national average, Shanxi might focus on coarse cereal cultivation. Shandong and Henan, whose agricultural efficiency exceeds both the FPMR average and the national level. Shandong might develop cash crops and Henan could maintain stable grain production.

## Electronic supplementary material

Below is the link to the electronic supplementary material.

Supplementary Material 1

Supplementary Material 2

## Data Availability

Data is provided within the manuscript or supplementary information files.
