# Supplementary material for: Extended exergy accounting of agricultural resources in China’s four provinces of mountains and rivers
Source: Sci Rep. 2025 Jul 1;15:22213. doi: 10.1038/s41598-025-06828-7 (PMC12217852; doi:10.1038/s41598-025-06828-7)
Supplement: Supplementary file 1 — Supplementary Material 1 [file 41598_2025_6828_MOESM1_ESM.docx]

## Table Supplementary Exergy coefficients of main resources and products

| Items | Exergy coefficient | Unit |
| --- | --- | --- |
| Coal | 22.2 | PJ/Mt |
| Coke | 29.9 | PJ/Mt |
| Oil and its products | 45.3 | PJ/Mt |
| Natural gas | 4.1 | PJ/10^8^ m^3^ |
| Electricity | 0.36 | PJ/10^8^ kwh |
| Rice | 15.8 | PJ/Mt |
| Wheat | 13.9 | PJ/Mt |
| Corn | 8.6 | PJ/Mt |
| Beans | 3.9 | PJ/Mt |
| Potatoes | 4.2 | PJ/Mt |
| Peanut | 24.6 | PJ/Mt |
| Rapeseed | 37 | PJ/Mt |
| Sesame | 39 | PJ/Mt |
| Other oil crops | 29.28 | PJ/Mt |
| Cotton | 16.4 | PJ/Mt |
| Hemp | 16.4 | PJ/Mt |
| Sugarcane and beet | 5 | PJ/Mt |
| Tea and tobacco | 10.7 | PJ/Mt |
| Vegetable | 1.9 | PJ/Mt |
| Fruit | 1.9 | PJ/Mt |
| Wood | 8 | PJ/Mm^3^ |
| Bamboo | 16 | PJ/Mt |
| Pork | 25.1 | PJ/Mt |
| Beef | 11.5 | PJ/Mt |
| Mutton | 16.1 | PJ/Mt |
| Milk | 4.9 | PJ/Mt |
| Egg | 6.2 | PJ/Mt |
| Silkworm cocoon | 4.5 | PJ/Mt |
| Wool | 3.8 | PJ/Mt |
| Aquatic products | 5.8 | PJ/Mt |
| Nitrogen fertilizer | 24.02 | PJ/Mt |
| Phosphate fertilizer | 8.49 | PJ/Mt |
| Potash fertilizer | 9.0 | PJ/Mt |
| Compound fertilizer | 13.84 | PJ/Mt |
| Chemical fiber | 18.5 | PJ/Mt |
| Plastic | 32.5 | PJ/Mt |
| Pesticide | 100 | PJ/Mt |
