# Supplementary material for: Extended exergy accounting of agricultural resources in China’s four provinces of mountains and rivers
Source: Sci Rep. 2025 Jul 1;15:22213. doi: 10.1038/s41598-025-06828-7 (PMC12217852; doi:10.1038/s41598-025-06828-7)
Supplement: Supplementary file 2 — Supplementary Material 2 [file 41598_2025_6828_MOESM2_ESM.docx]

Supplementary Information

The calculation of E_L_ and E_K_

First, we calculated the E_L_ of China.

E_L_ of China: E_L_=f×e_surv_×N_h_=(0.75/0.055) ×10^7^×1.4×10^9^×365=6.97× 10^19^ J

M2/(s×N_w_×W)=8.2×10^13^/(7.4×10^4^×7.9×10^8^×2000)=7.01× 10^-4^

Second, we calculated the E_L_ of the agricultural sector.

Based on the China Labour Statistical Yearbook, the labour and average salary in China were 1.8×10^8^ and 7.4×10^4^, and that of agricultural sector were 2.6 ×10^6^ and 3.6×10^4^

E_L_(AG)=s_AG_×N_WAG_×E_L_/(s×N_w_)= 3.6×10^4^×2.6 ×10^6^×6.97× 10^19^ J /(7.4×10^4^×1.8×10^8^)

=4.95×10^17^ J=495 PJ

Third, we got the E_L_ of the agricultural sector in the four provinces.

We got labour and average salary in the four provinces from the China Labour Statistical Yearbook, similar to the step two, we got the E_L_ of the agricultural sector in four provinces. Take the Hebei province as the example, the labour and average salary were 3.5×10^4^ and 2.3×10^4^.

E_L_(Hebei)= 3.5×10^4^×2.3×10^4^×495PJ/(3.6×10^4^×2.6 ×10^6^)=4.25PJ

Forth, we calculated the E_K_ in the FPMR and took the Hebei province as the example.

E_K_=7.01× 10^-4^×4.25×10^15^J=0.3×10^12^J=0.3TJ

In the same way, we got the E_L_ and E_K_ in other three provinces and the FPMR.

The calculation of E_R_

According to former research ^16,68^, we got the coefficients of different emissions and took the Hebei province as the example.

Hogs was 19.58 million according to the China Statistical Yearbook and animal wastes includes three parts:

Dry dung: 19.58×398×17.8 PJ/Mt×0.75× 0.001=104.02PJ

COD: 19.58×10.33×13.6 PJ/Mt×0.75× 0.001=2.06 PJ

NH_3_-N: 19.58×7.68×19.8PJ/Mt×0.75× 0.001=2.23 PJ

Large animals (horses, donkeys, cattle and mules), poultry and sheep were in the same method.

CO_2_

Coal 95.32×1.7=1.62 Mt

Oil 154.99×3=4.64Mt

Natural gas 3.1×2.16=0.67Mt

The exergy of CO_2_: (1.62+4.64+0.67) Mt×0.45 PJ/Mt =3.11PJ

The exergy of N_2_O and CH_4_ were in the same method according the research^16,68^

Residues of fertilizers, pesticides and plastic mulch

Nitrogen fertilizer: 140.3×24.02 PJ/Mt×0.35×0.01=11.79 PJ

Phosphate fertilizer 43.7×8.49 PJ/Mt×0.35×0.01=1.29 PJ

Potash fertilizer 26.8× 9 PJ/Mt×0.35×0.01=0.84 PJ

Compound fertilizer 111.2×13.84 PJ/Mt×0.35×0.01=5.39 PJ

Pesticides: 7.76×100.42 PJ/Mt×0.37×0.01=2.88 PJ

Plastic mulch: 12.8×32.5 PJ/Mt×0.42×0.01=1.75PJ
